# Supplementary material for: The Impact of Fast Radiation on the Phylogeny of Bactrocera Fruit Flies as Revealed by Multiple Evolutionary Models and Mutation Rate-Calibrated Clock
Source: Insects. 2022 Jun 30;13(7):603. doi: 10.3390/insects13070603 (PMC9319077; doi:10.3390/insects13070603)

Figure S1. Phylogeny of *Bactrocera* obtained by RAxML using a Maximum Likelihood analysis on the concatenated codon alignments of 110 orthologous nuclear genes (189,891 nt). Support at nodes is given as bootstrap values.

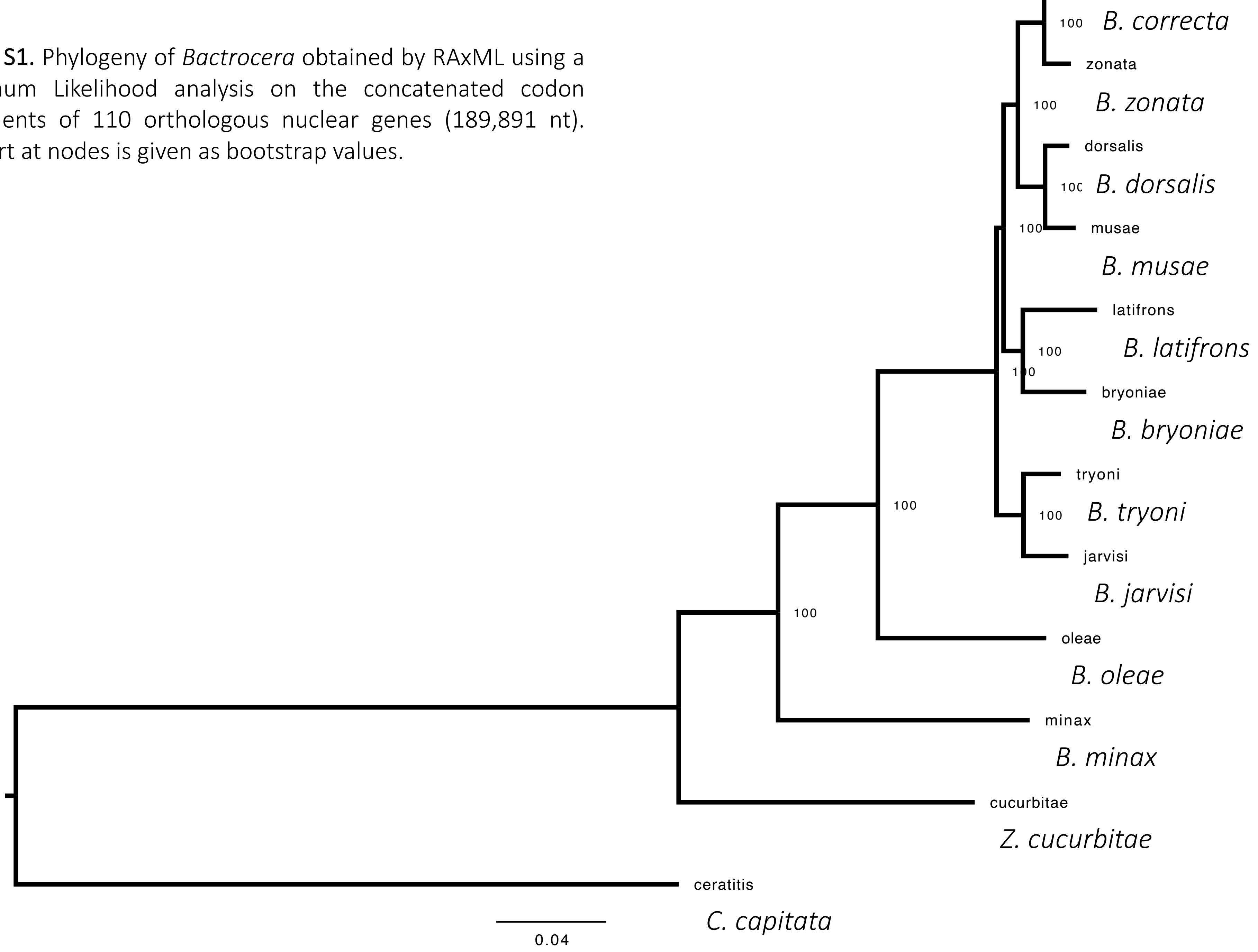

Figure S2. Phylogeny of *Bactrocera* obtained by Beast2 using a Bayesian analysis on the concatenated amino acidic alignments (63,297 aa). Support at nodes is given as posterior probabilities.

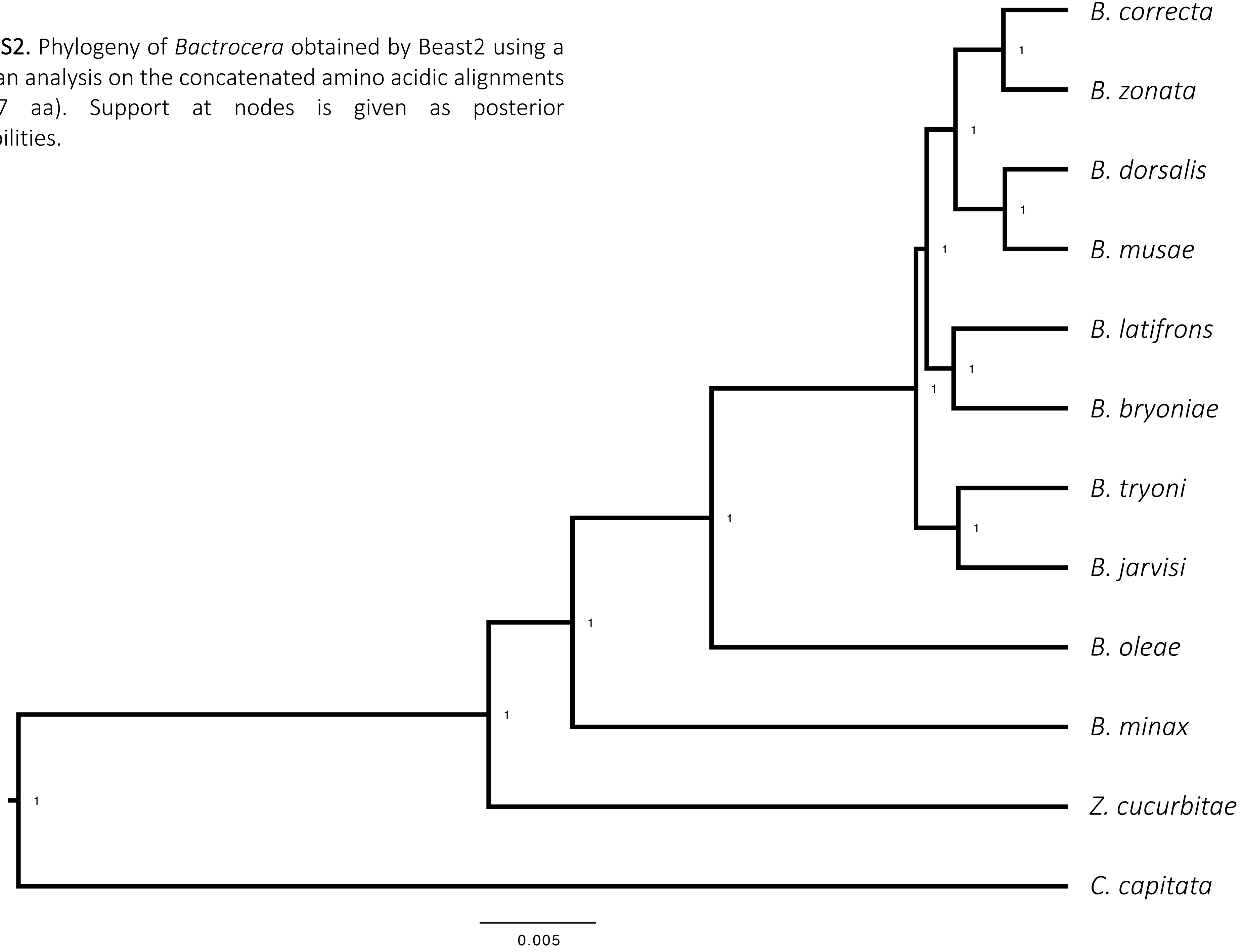

Figure S3. Phylogeny of *Bactrocera* obtained by Beast2 using a Bayesian analysis on the concatenated codon alignments of 110 orthologous nuclear genes (189,891 nt). Support at nodes is given as posterior probabilities.

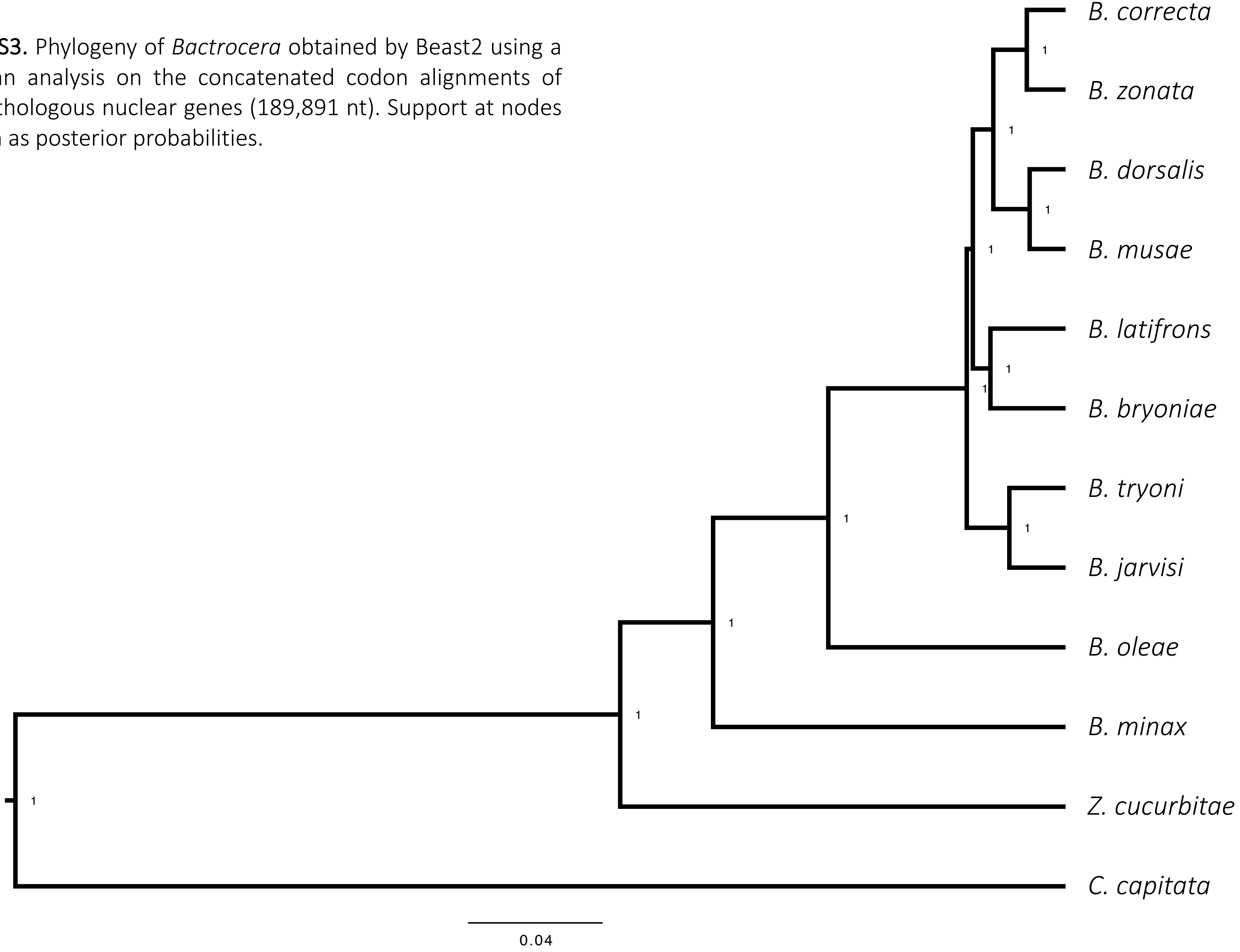

Figure S4. Phylogeny of *Bactrocera* obtained by PhyloBayes using a Bayesian analysis on the concatenated amino acidic alignments (63,297 aa). Support at nodes is given as posterior probabilities.

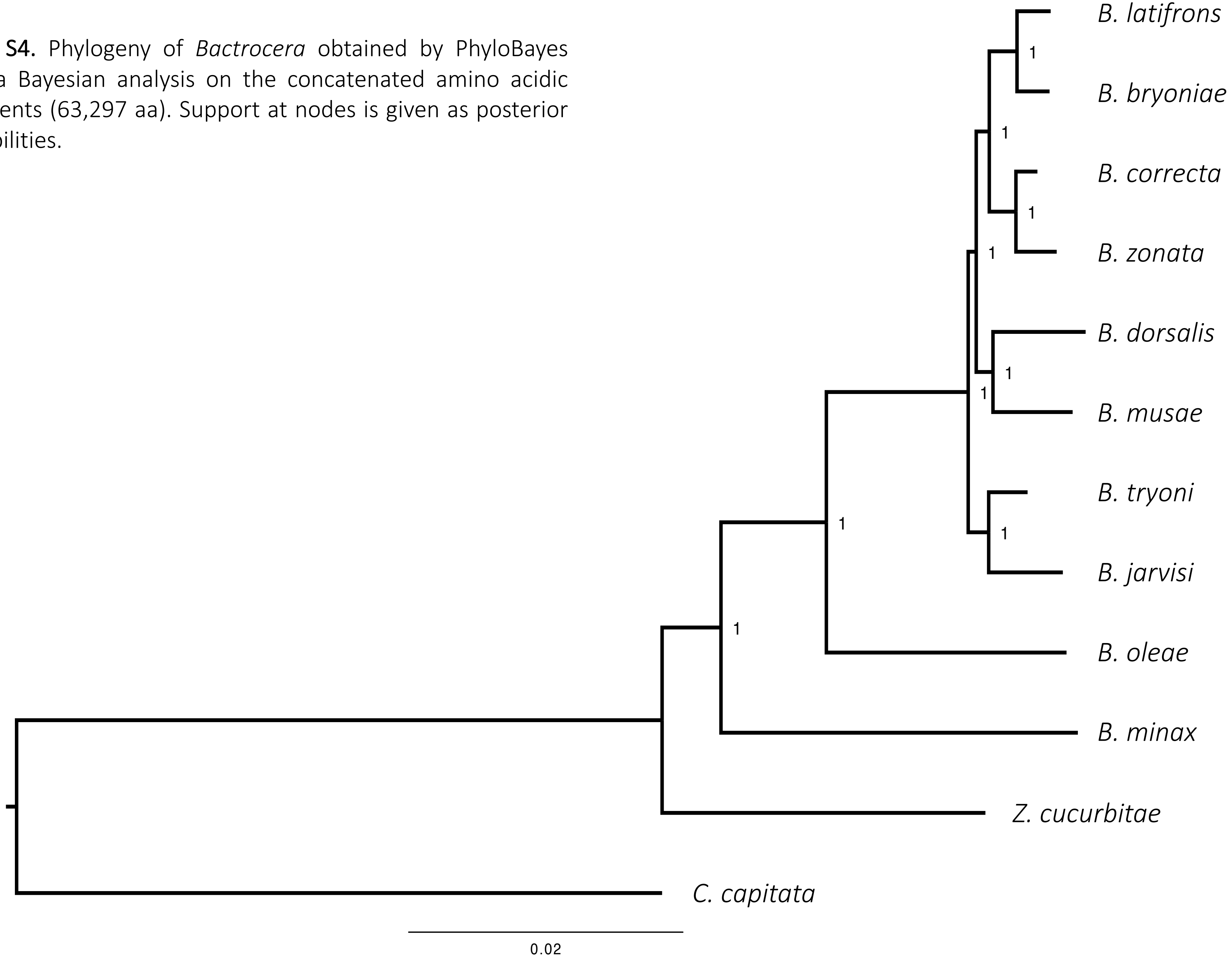

Figure S5. Phylogeny of *Bactrocera* obtained by Beast2 using a Bayesian analysis on the 4-fold degenerate sites of the concatenated alignments of 110 orthologous nuclear genes (24,885 nt). Support at nodes is given as posterior probabilities.

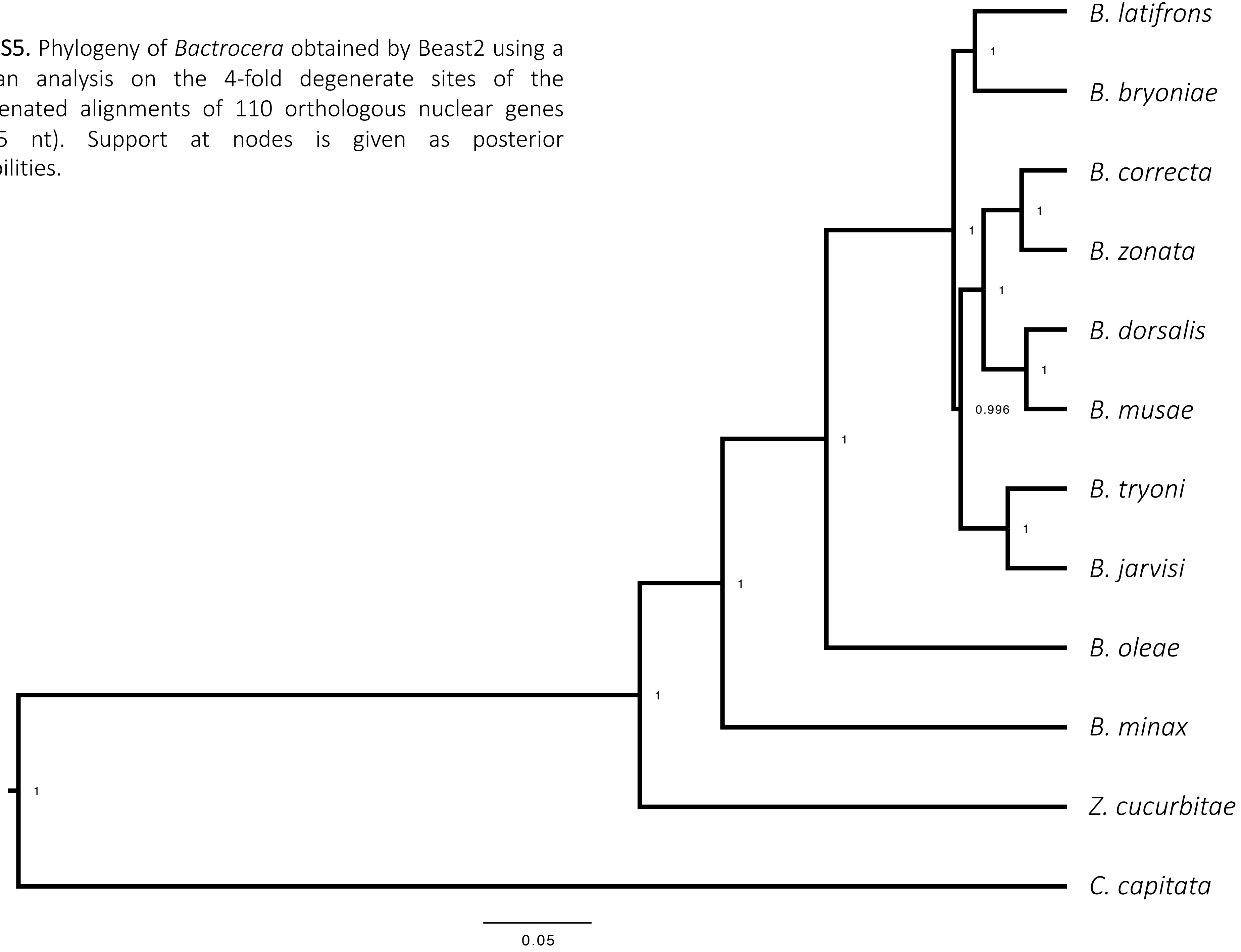

Figure S6. Phylogeny of *Bactrocera* obtained by PhyloBayes using a Bayesian analysis on the 4-fold degenerate sites of the concatenated alignments of 110 orthologous nuclear genes (24,885 nt). Support at nodes is given as posterior probabilities.

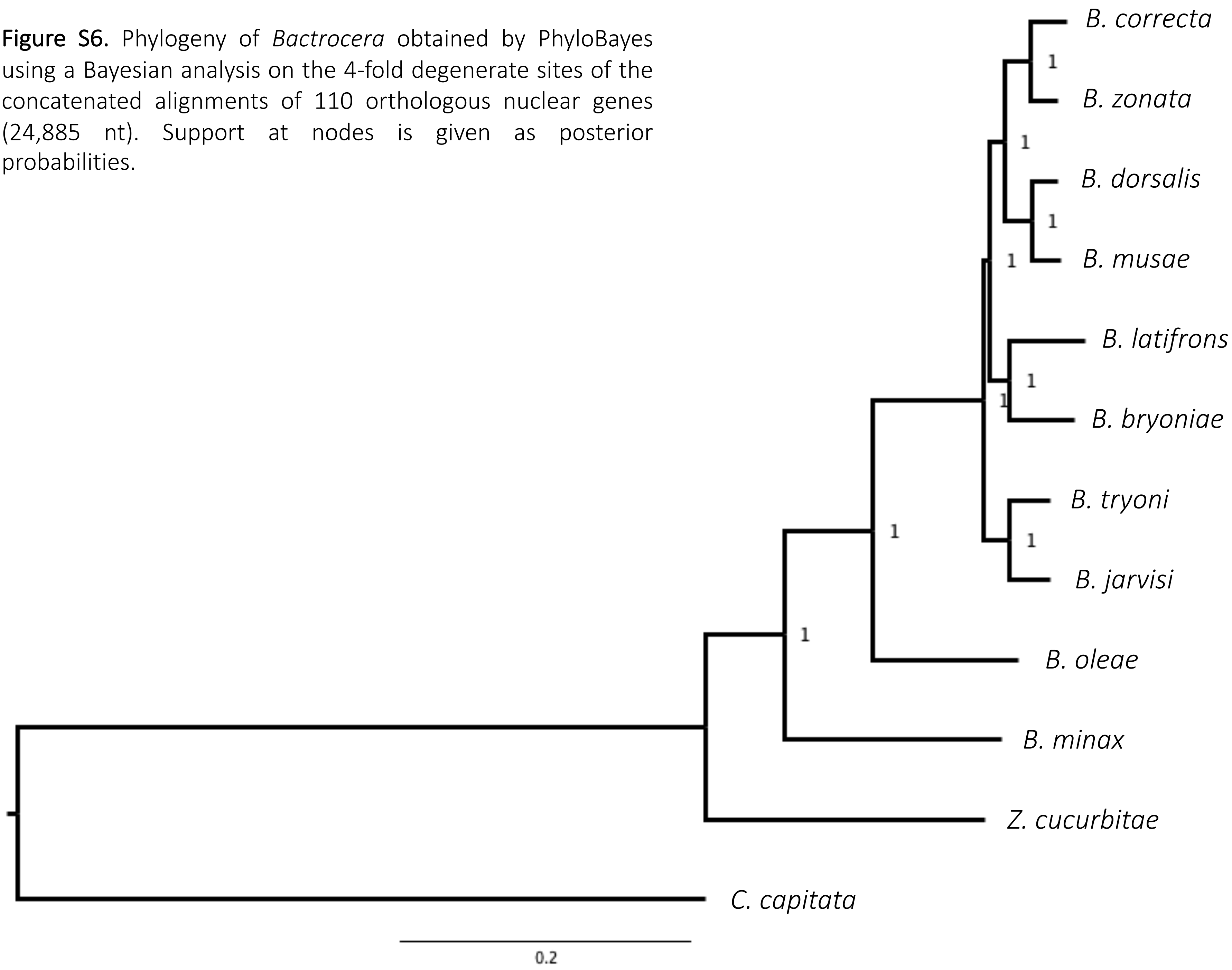

Figure S7. Phylogeny of *Bactrocera* obtained by RAxML using a Maximum Likelihood analysis on the 4-fold degenerate sites of the concatenated alignments of 110 orthologous nuclear genes (24,885 nt). Support at nodes is given as bootstrap values.

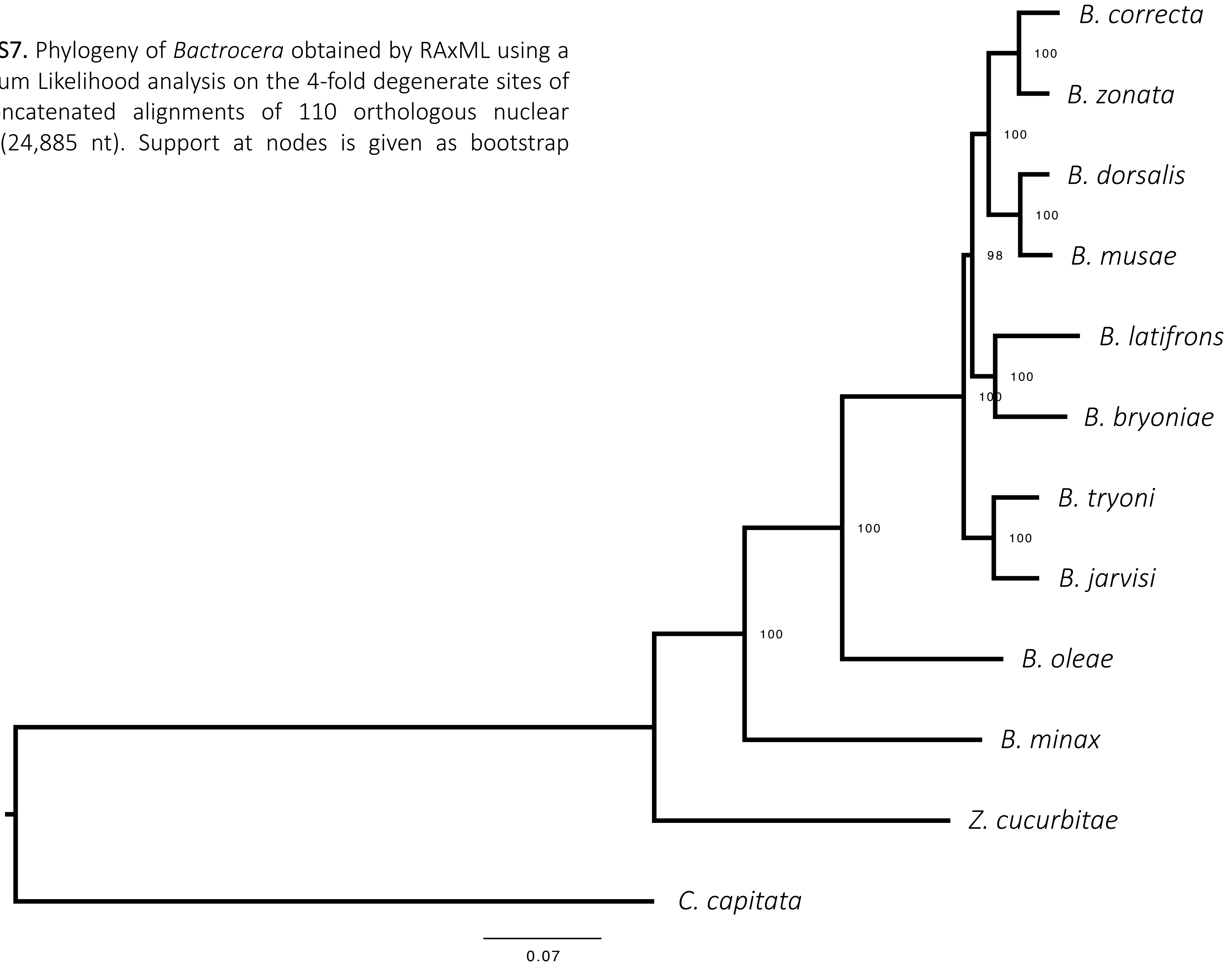

Figure S8. Multi-locus coalescent-aware phylogenesis of *Bactrocera* inferred from 110 orthologous nuclear genes using ASTRAL. Analyses are based on all single ML gene trees obtained by RAxML using the aminoacidic alignments. Bootstrap values were estimated by performing 100 multi-locus bootstrap replicates.

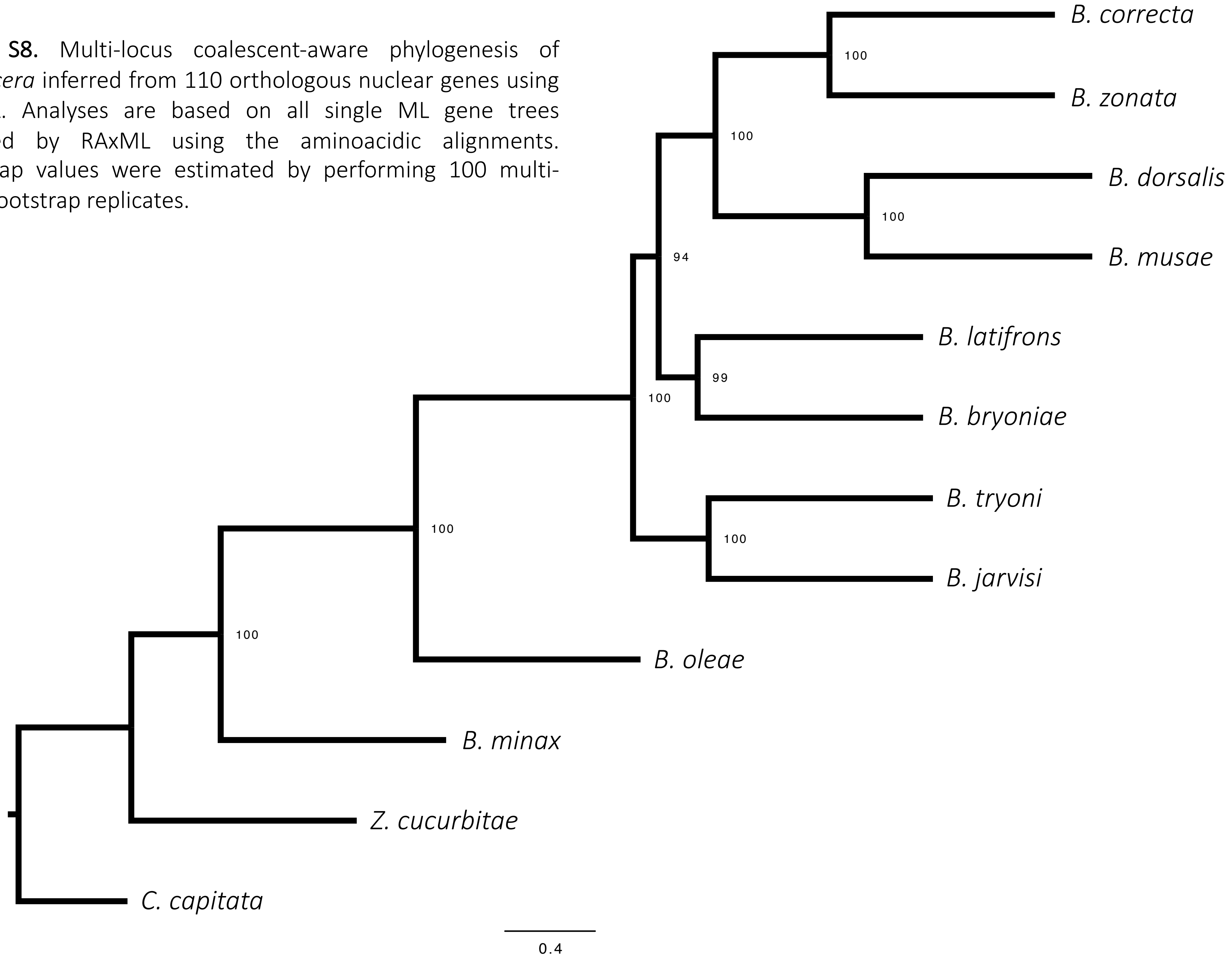

Figure S9. Multi-locus coalescent-aware phylogenesis of *Bactrocera* inferred from 110 orthologous nuclear genes using ASTRAL. Analyses are based on all single ML gene trees obtained by RAxML using the aminoacidic alignments. Bootstrap values were estimated by performing 100 gene+site resampling.

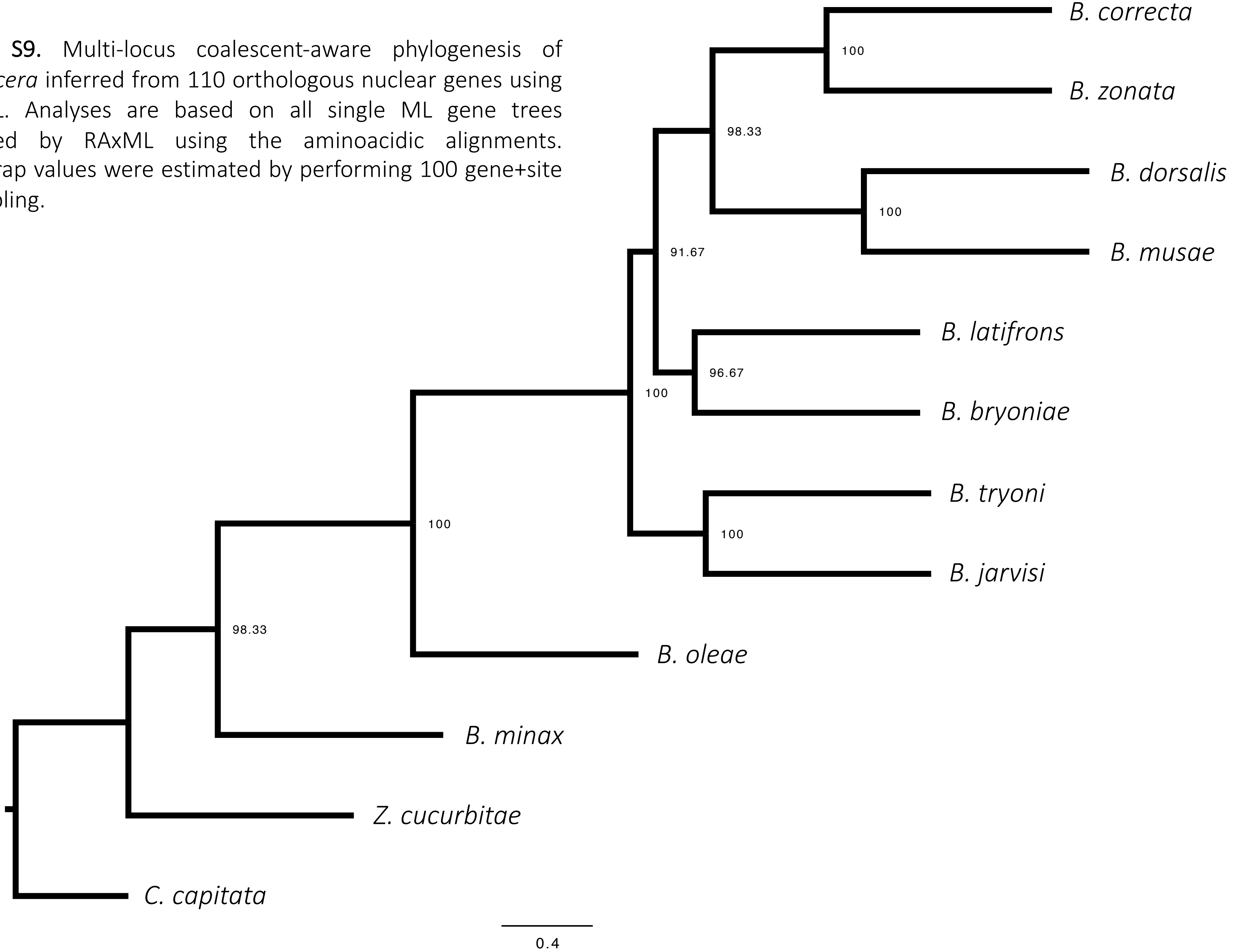

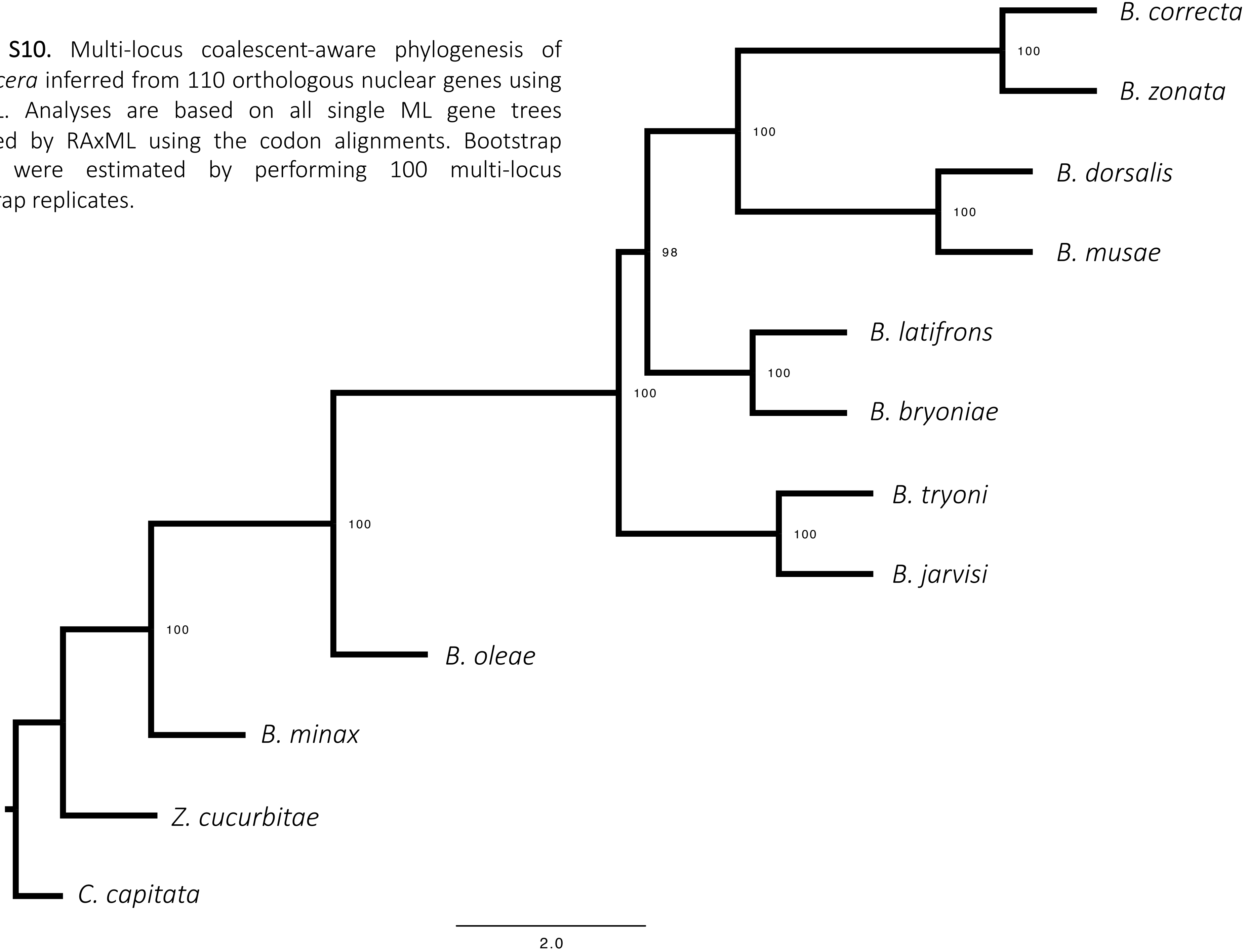

Figure S10. Multi-locus coalescent-aware phylogenesis of *Bactrocera* inferred from 110 orthologous nuclear genes using ASTRAL. Analyses are based on all single ML gene trees obtained by RAxML using the codon alignments. Bootstrap values were estimated by performing 100 multi-locus bootstrap replicates.

Figure S11. Multi-locus coalescent-aware phylogenesis of *Bactrocera* inferred from 110 orthologous nuclear genes using ASTRAL. Analyses are based on all single ML gene trees obtained by RAxML using the codon alignments. Bootstrap values were estimated by performing 100 gene+site resampling.

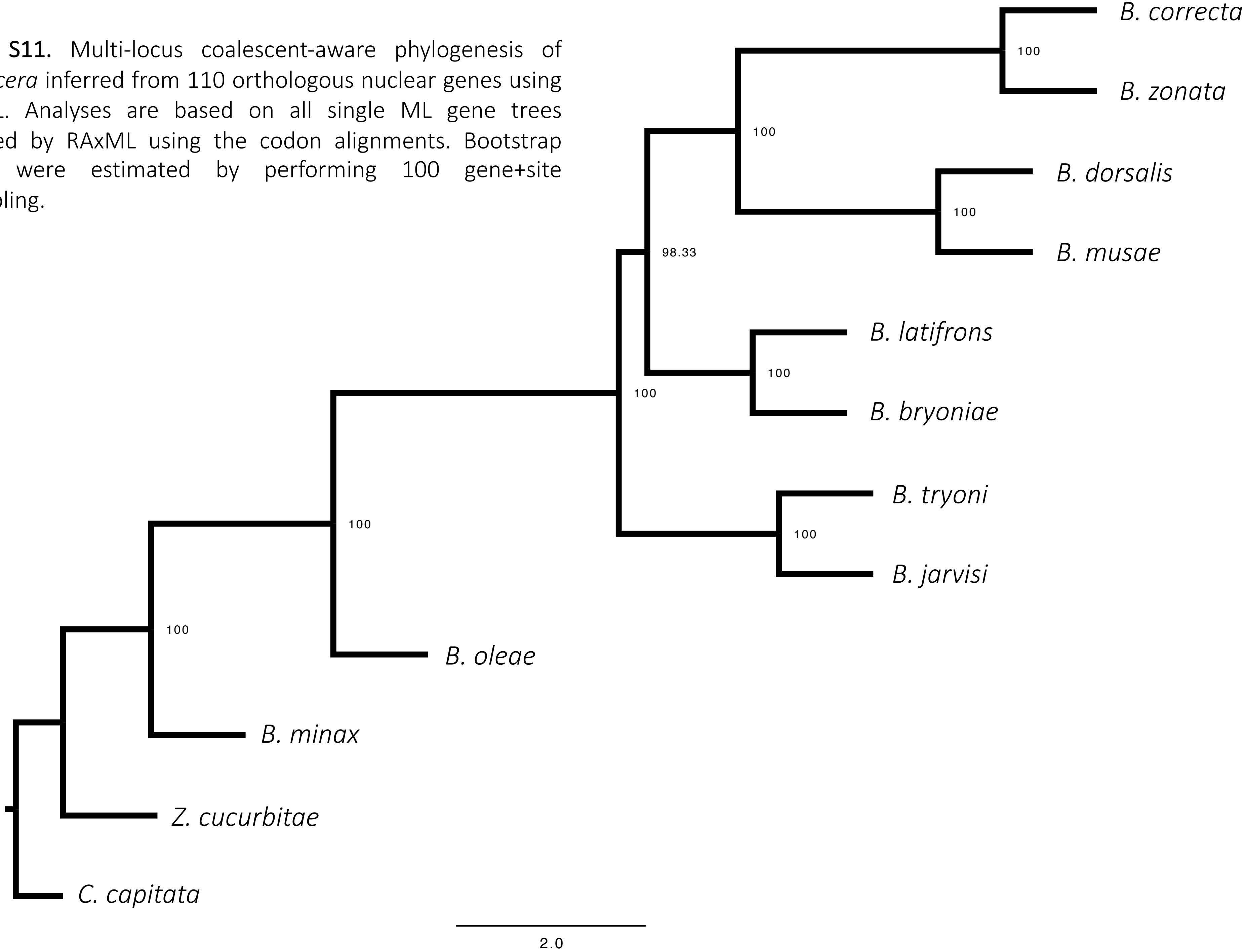

Figure S12. Multi-locus phylogenesis of *Bactrocera*. Bayesian analyses were obtained by StarBeast2 for each of the 110 amino acidic orthologous genes alignments employing a multispecies coalescent method to estimate the species tree. The site models were linked across the genes.

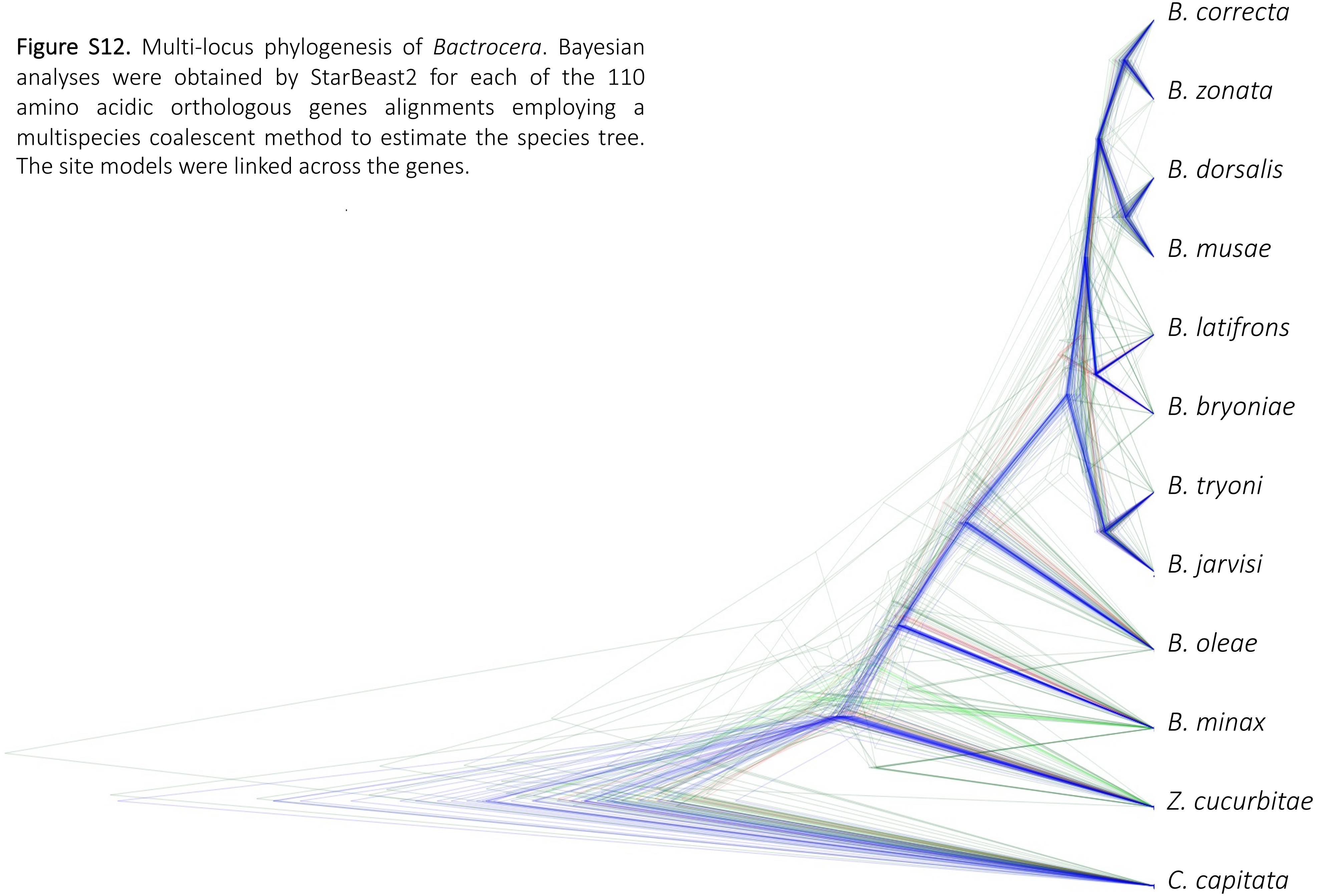

Figure S13. Multi-locus phylogenesis of *Bactrocera*. Bayesian analyses were obtained by StarBeast2 for each of the 110 codon orthologous genes alignments employing a multispecies coalescent method to estimate the species tree. The site models were linked across the genes.

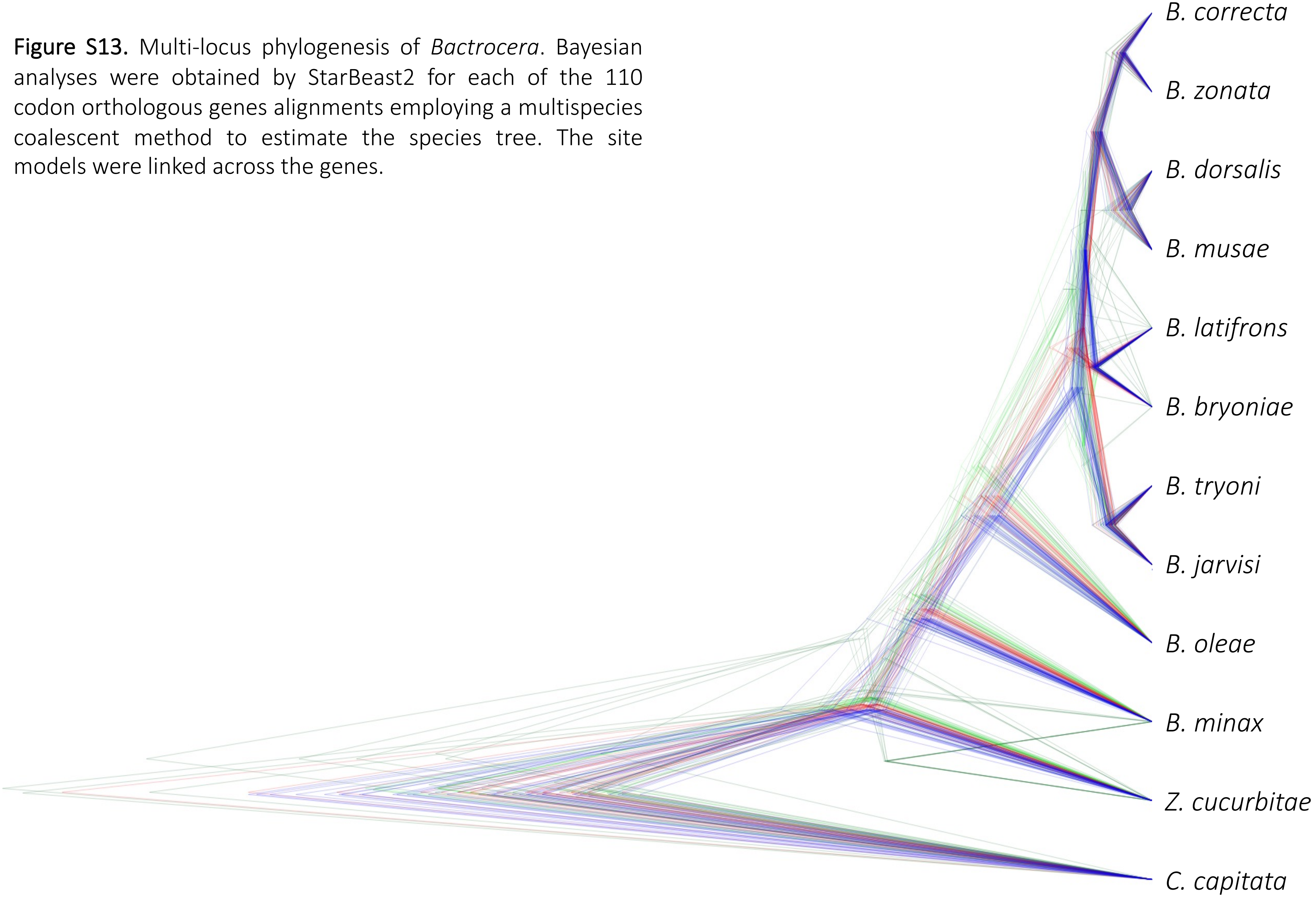

Figure S14. Molecular time tree of *Bactrocera* obtained by Beast2 using the 4-fold degenerate sites of the concatenated alignments of 110 orthologous nuclear genes (24,885 nt). We used a mutation rate log-normally distributed as prior, a log-normal clock and a Birth-Death model. Mean and 95% confidence interval of the inferred age (corresponding to the blue bars) are reported for each node.

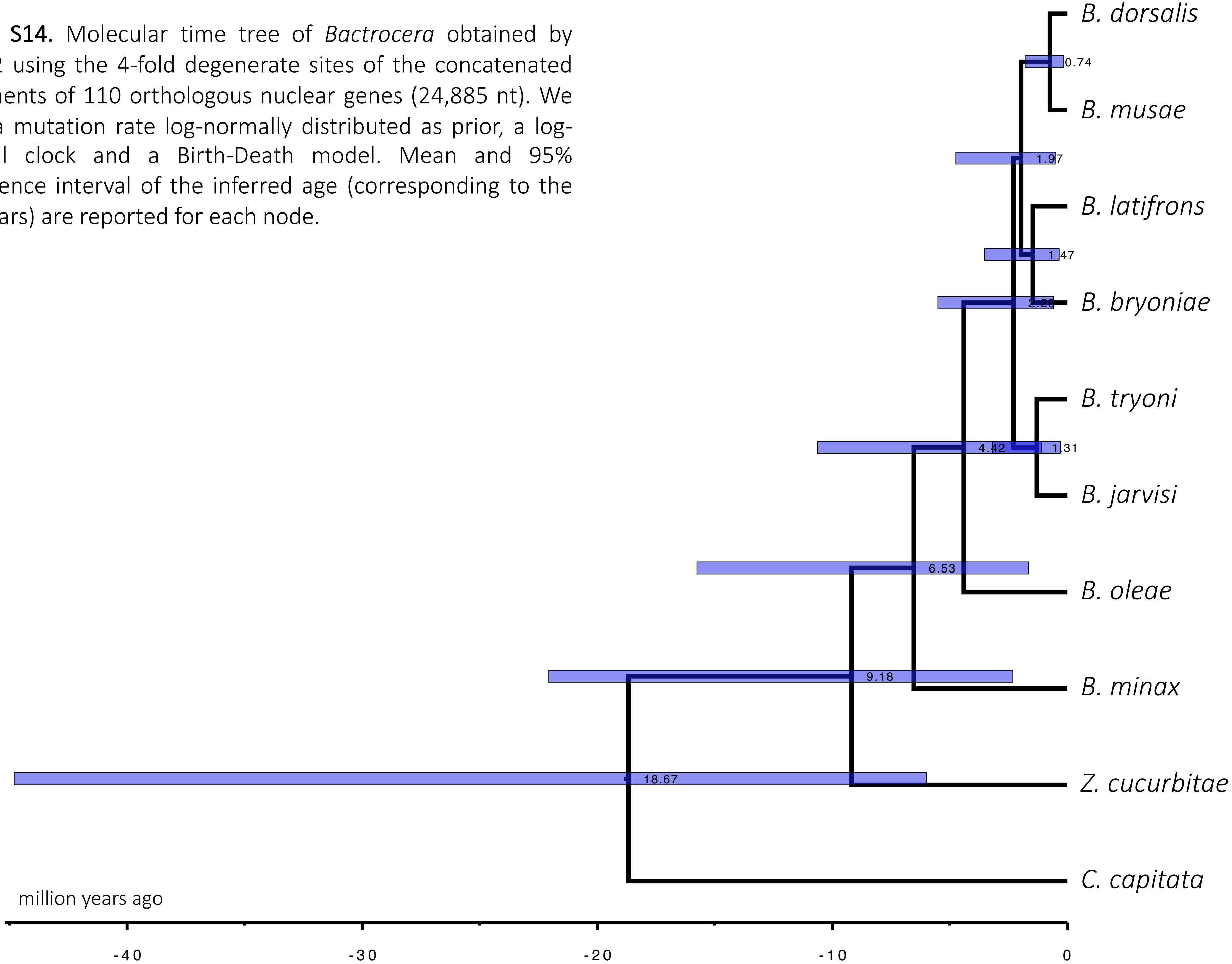

Figure S15. Molecular time tree of *Bactrocera* obtained by Beast2 using the 4-fold degenerate sites of the concatenated alignments of 110 orthologous nuclear genes (24,885 nt). We used a mutation rate log-normally distributed as prior, a strict clock and a Birth-Death model. Mean and 95% confidence interval of the inferred age (corresponding to the blue bars) are reported for each node.

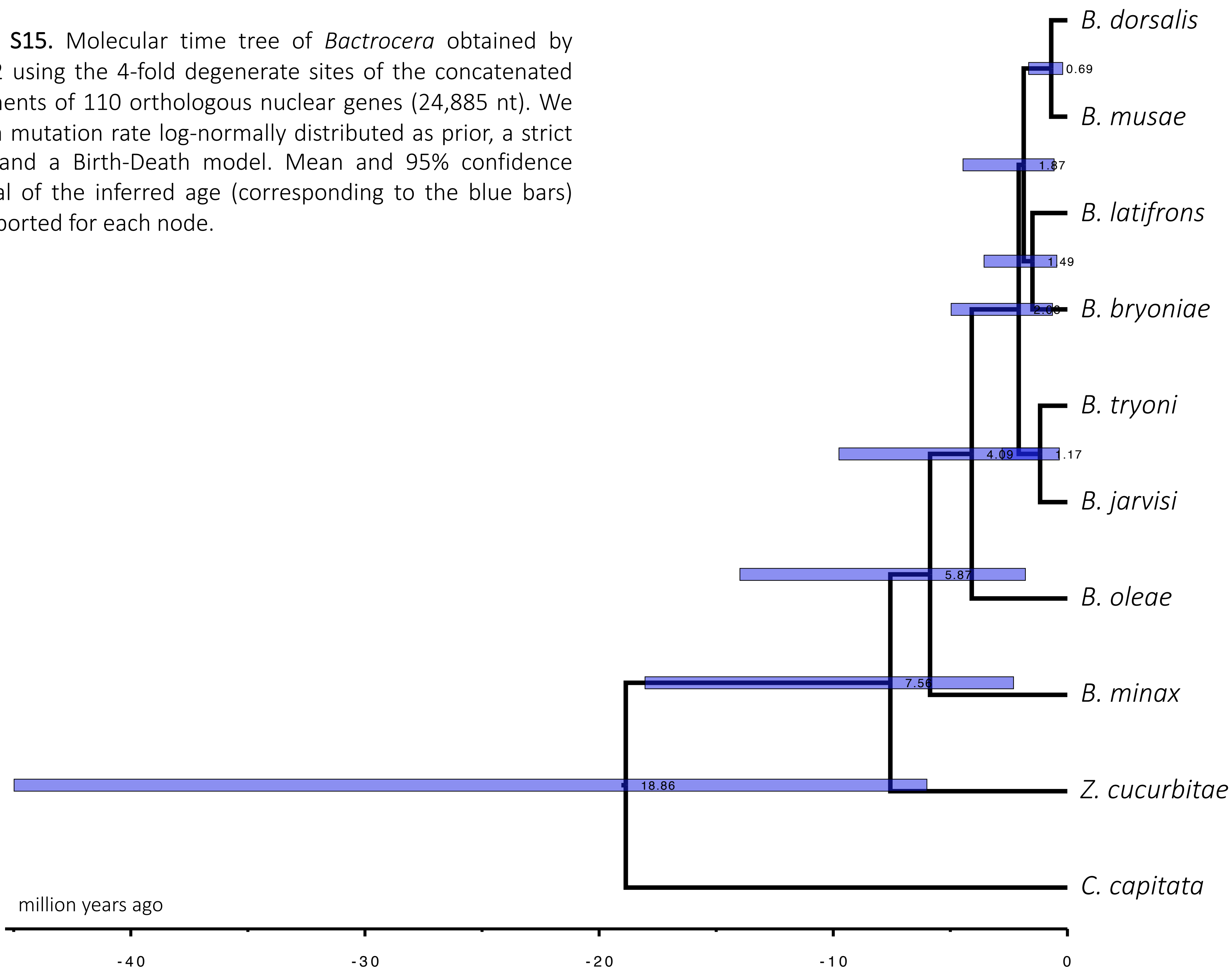

Figure S16. Molecular time tree of *Bactrocera* obtained by Beast2 using the 4-fold degenerate sites of the concatenated alignments of 110 orthologous nuclear genes (24,885 nt). We used a mutation rate log-normally distributed as prior, a log-normal clock and a Yule model. Mean and 95% confidence interval of the inferred age (corresponding to the blue bars) are reported for each node.

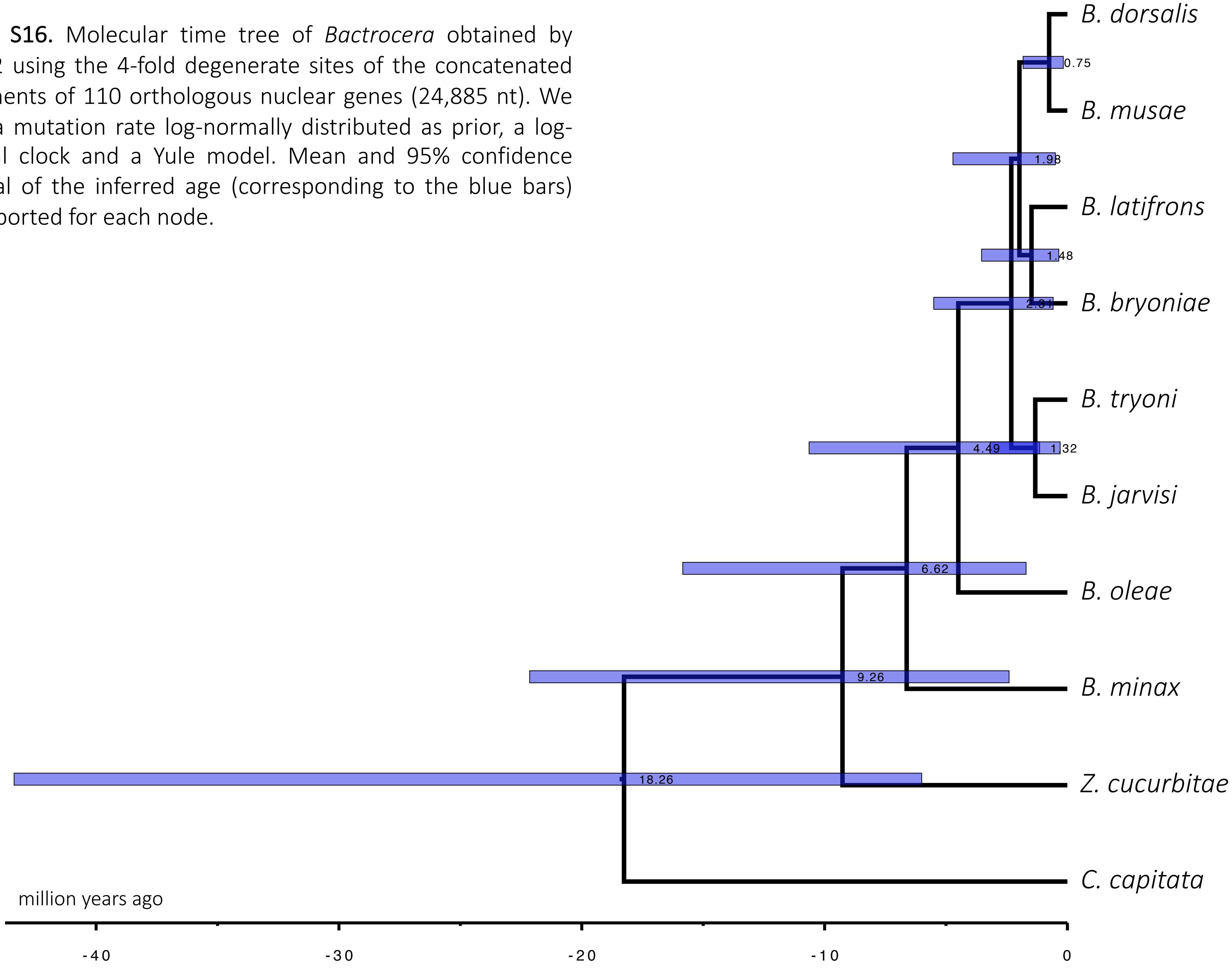

Figure S17. Molecular time tree of *Bactrocera* obtained by Beast2 using the 4-fold degenerate sites of the concatenated alignments of 110 orthologous nuclear genes (24,885 nt). We used a mutation rate log-normally distributed as prior, a strict clock and a Yule model. Mean and 95% confidence interval of the inferred age (corresponding to the blue bars) are reported for each node.

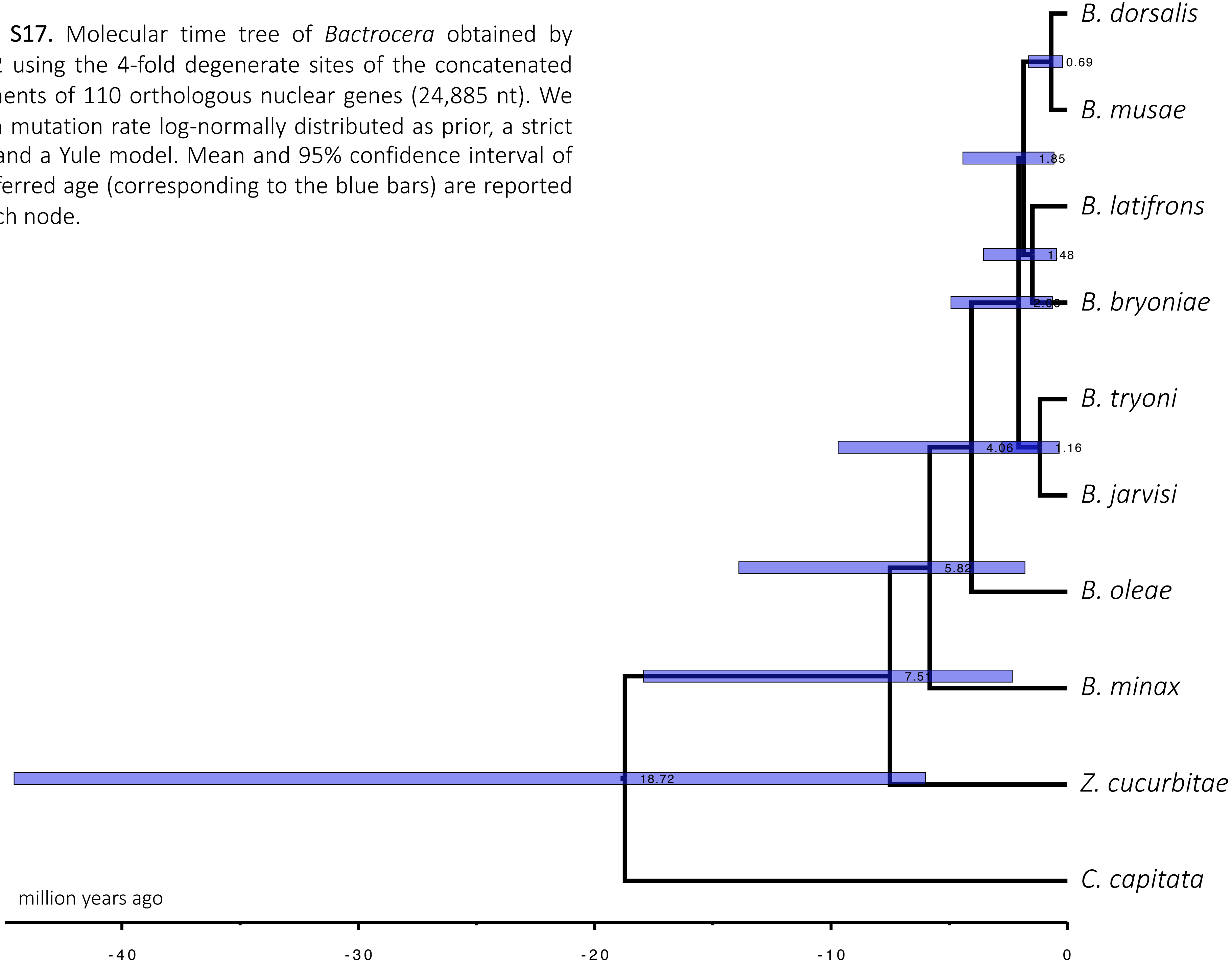

Figure S18. Molecular time tree of *Bactrocera* obtained by Beast2 using the 4-fold degenerate sites of the concatenated alignments of 110 orthologous nuclear genes (24,885 nt). We used a mutation rate normally distributed as prior, a log-normal clock and a Birth-Death model. Mean and 95% confidence interval of the inferred age (corresponding to the blue bars) are reported for each node.

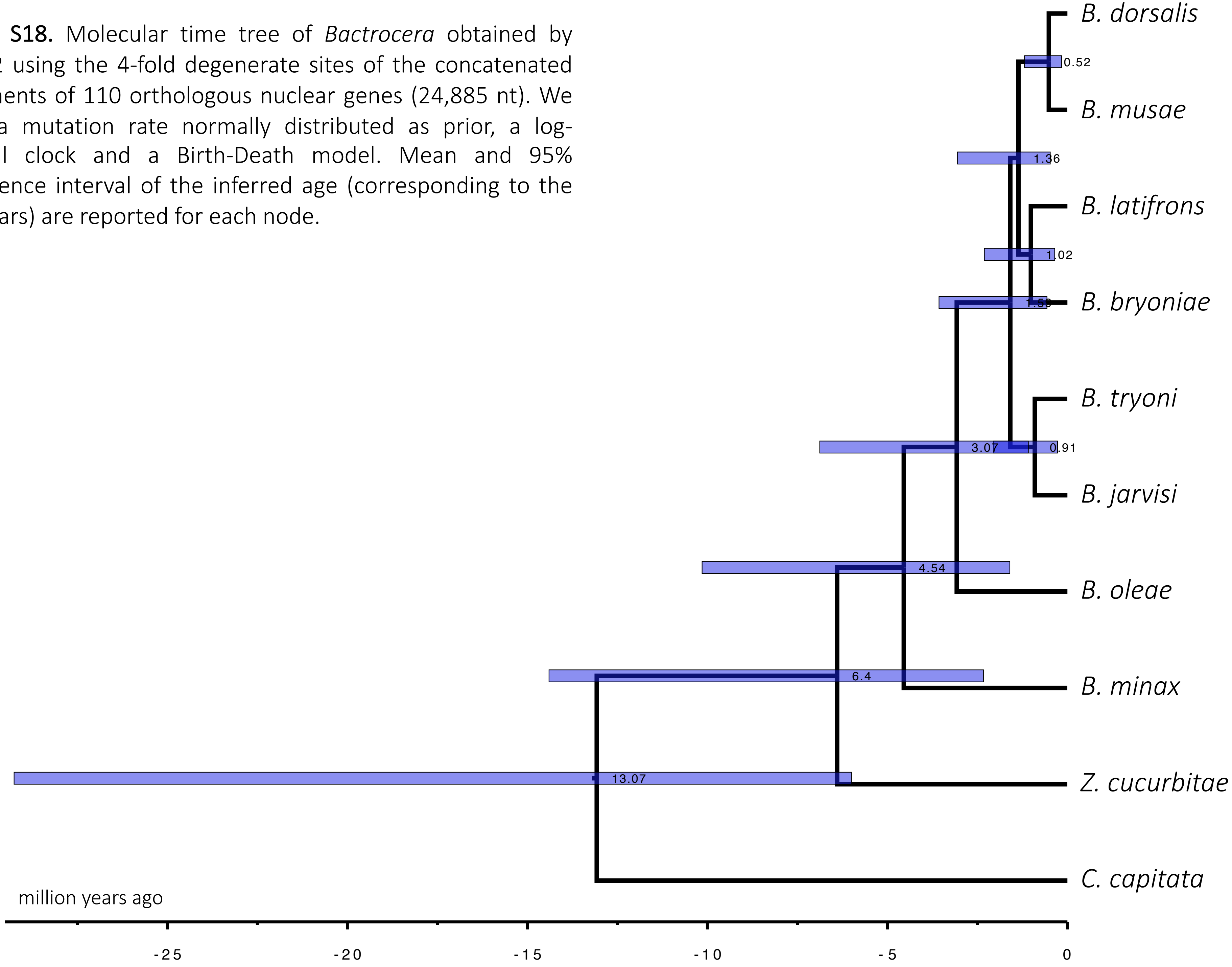

Figure S19. Molecular time tree of *Bactrocera* obtained by Beast2 using the 4-fold degenerate sites of the concatenated alignments of 110 orthologous nuclear genes (24,885 nt). We used a mutation rate normally distributed as prior, a strict clock and a Birth-Death model. Mean and 95% confidence interval of the inferred age (corresponding to the blue bars) are reported for each node.

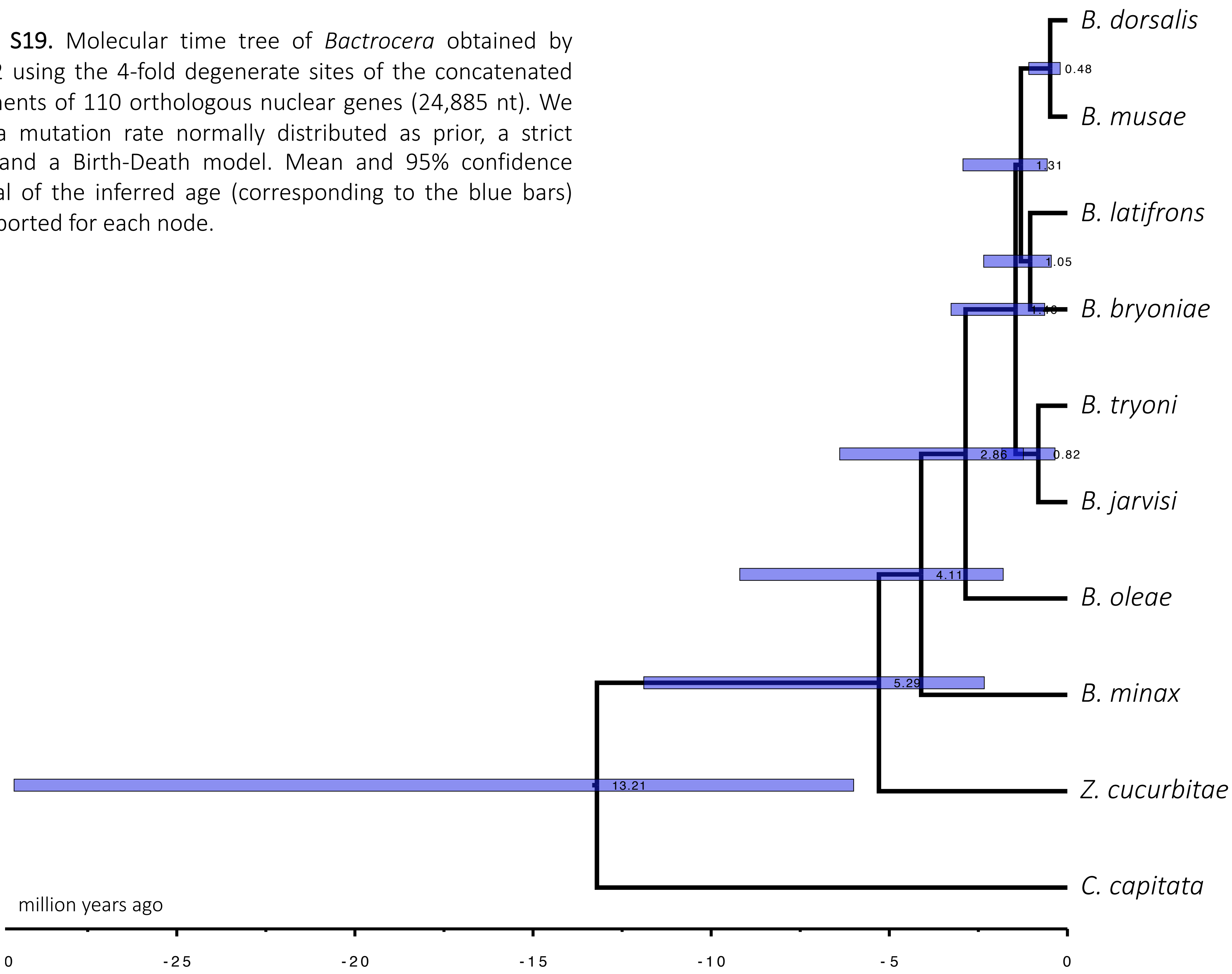

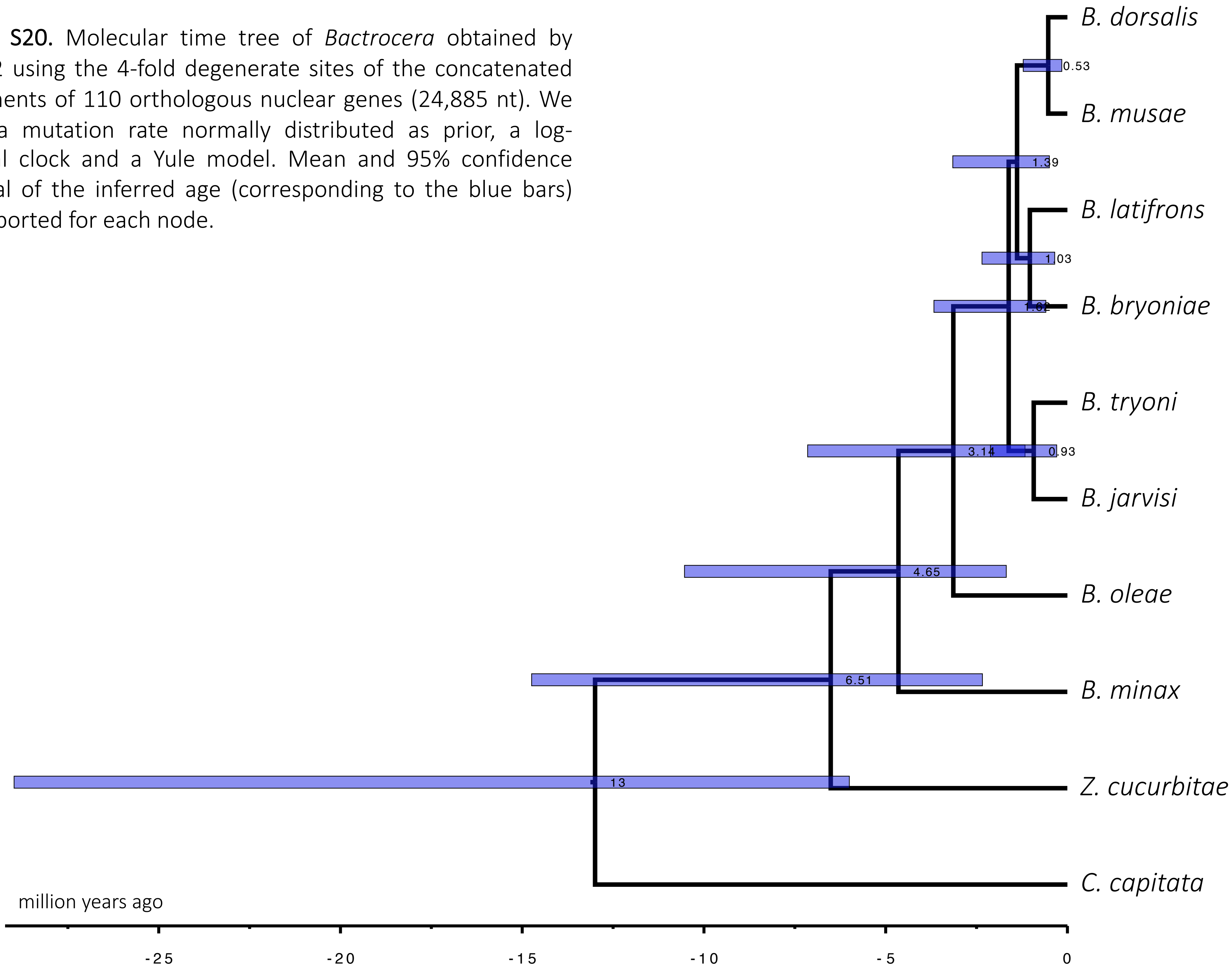

Figure S20. Molecular time tree of *Bactrocera* obtained by Beast2 using the 4-fold degenerate sites of the concatenated alignments of 110 orthologous nuclear genes (24,885 nt). We used a mutation rate normally distributed as prior, a log-normal clock and a Yule model. Mean and 95% confidence interval of the inferred age (corresponding to the blue bars) are reported for each node.

Figure S21. Molecular time tree of *Bactrocera* obtained by Beast2 using the 4-fold degenerate sites of the concatenated alignments of 110 orthologous nuclear genes (24,885 nt). We used a mutation rate normally distributed as prior, a strict clock and a Yule model. Mean and 95% confidence interval of the inferred age (corresponding to the blue bars) are reported for each node.

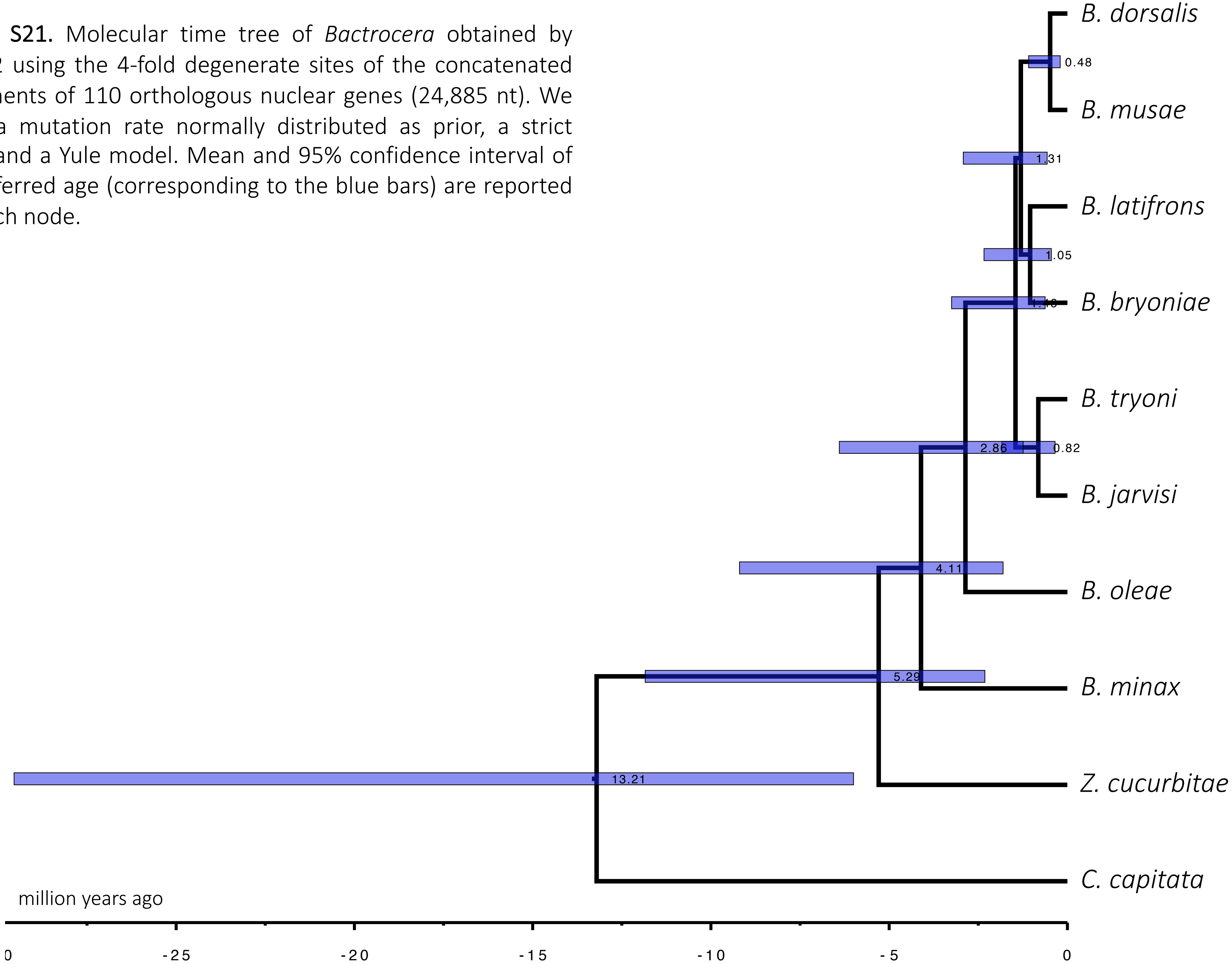

Supplement: Supplementary file 1 [file insects-13-00603-s001.zip › Figures S1-S21.pdf]
