# Supplementary material for: The Impact of Fast Radiation on the Phylogeny of Bactrocera Fruit Flies as Revealed by Multiple Evolutionary Models and Mutation Rate-Calibrated Clock
Source: Insects. 2022 Jun 30;13(7):603. doi: 10.3390/insects13070603 (PMC9319077; doi:10.3390/insects13070603)
Supplement: Supplementary file 1 [file insects-13-00603-s001.zip › Table S1.pdf]

Table S1. Results of the model selection of the different BEAST analyses used to estimate divergence times.

			Model 2							
			Mut-normal				*Mut-lognormal*			
			yule_strict	*yule_rlxln*	*bd_strict*	*bd_rlxln*	*yule_strict*	*yule_rlxln*	*bd_strict*	*bd_rlxln*
Model 1	*Mut-normal*	***yule_strict***	-	62.2	*	54.3	9.2	59.6	#	57.2
		yule_rlxln	#	-	#	#	#	#	#	#
		bd_strict	#	58.6	-	50.7	-	55.9	#	53.5
		bd_rlxln	#	7.9	#	-	#	5.2	#	*
	Mut-lognormal	***yule_strict***	#	53.1	#	45.2	-	50.4	#	48
		yule_rlxln	#	*	#	#	#	-	#	#
		bd_strict	**6.1**	**68.3**	**9.8**	**60.4**	**15.3**	**65.7**	-	**63.3**
		bd_rlxln	#	*	#	#	#	*	#	-

Bayes Factors (BF) were estimated comparing eight BEAST models that combined a mutation prior with either a normal (*Mut-normal*) or lognormal (*Mut-lognormal*) distribution, either a Yule (*yule*) or a Birth-Death (*bd*) model, and either a *strict* or a LOGN relaxed (*rlxln*) clock. Models were compared in a pairwise fashion, by first estimating their Marginal Likelihoods (mL) and corresponding Standard Deviation (SD) and then calculating the Bayes Factors as $BF = mL_1 - mL_2$, where model 1 and model 2 are those given in the respective row and column. We only report BF values that satisfied the conditions $BF > 0$ and $BF-(SD_1+SD_2) > 0$, where SD_1 and SD_2 are the SD values estimated for model 1 and 2. Highlighted on bold are the values for the model (*Mut-lognormal + bd + strict*) favored over all other seven models. # = $BF < 0$; * = $BF-(SD_1+SD_2) < 0$.
