# Supplementary material for: The Impact of Fast Radiation on the Phylogeny of Bactrocera Fruit Flies as Revealed by Multiple Evolutionary Models and Mutation Rate-Calibrated Clock
Source: Insects. 2022 Jun 30;13(7):603. doi: 10.3390/insects13070603 (PMC9319077; doi:10.3390/insects13070603)
Supplement: Supplementary file 1 [file insects-13-00603-s001.zip › Table S2.pdf]

TableS2. SRA databases used to reconstruct the transcriptome of five *Bactrocera* species.

**Species**	**SRA databases** (https://www.ncbi.nlm.nih.gov/sra)
Bactrocera bryoniae	SRX2791703
Bactrocera correcta	SRX2013592, SRX2013591, SRX2013590, SRX2013589, SRX2372821, SRX2372818, SRX2372817
Bactrocera jarvisi	SRX2791705, SRX697442, SRX697441, SRX697440, SRX697437, SRX697435, SRX697434, SRX697431, SRX697428
Bactrocera musae	SRX2791704
Bactrocera zonata	SRX2016849, SRX2016848, SRX2016847, SRX2016846
